# Immunization coverage of 12–23 months old children and associated factors in Jigjiga District, Somali National Regional State, Ethiopia

**DOI:** 10.1186/1471-2458-14-865

**Published:** 2014-08-22

**Authors:** Abdi Nur Mohamud, Amsalu Feleke, Walelegn Worku, Manay Kifle, Hardeep Rai Sharma

**Affiliations:** Jigjiga Health Science College, Somali National Regional State, Jigjiga, Ethiopia; Health Service Management and Health Economics Department, College of Medicine and Health Science, University of Gondar, PO Box No. 196, Gondar, Ethiopia; Department of Environmental and Occupational Health and Safety, College of Medicine and Health Science, University of Gondar, PO Box No. 196, Gondar, Ethiopia; Institute of Environmental Studies, Kurukshetra University, Kurukshetra, Haryana India

**Keywords:** Immunization coverage, Vaccination, Children, Ethiopia

## Abstract

**Background:**

Immunization coverage in Ethiopia is less than the herd immunity level desired to prevent the spread of eight target diseases targeted by the World Health Organization’s Expanded Program of Immunization. In particular, the Somali region of the country still has by far the lowest level of immunization coverage. The objective of this study was to measure the immunization coverage of 12–23 months old children and associated factors in the urban and rural areas of Jigjiga district.

**Methods:**

A community based cross-sectional survey was conducted in 582 households with 12–23 months old children in two urban and four rural wards. The data were collected from mothers or caregivers through interviews based on pre-tested and structured questionnaires and from the review of vaccination cards. Data were processed using SPSS version 16. To identify factors associated with the immunization status of children, bivariate and multiple logistic regression analyses were worked out and the Hoshmer and Lemeshow’s goodness-of-fit was used to assess the fitness of multiple logistic regression model.

**Results:**

Three–fourth (74.6%) of the children surveyed were ever vaccinated, whereas 36.6% were fully vaccinated. The immunization coverage rate from card assessment for Bacillus Calmette-Guérin was 41.8%, while for Oral Polio Vaccine Zero, Oral Polio Vaccine One /Pentavalent1, Oral Polio Vaccine Two /Pentavalent2, Oral Polio Vaccine Three /Pentavalent3, and measles were 10.4%, 41.1%, 33.9%, 27.5%, and 24.9%, respectively. Maternal literacy (AOR = 3.06, 95% CI = 1.64, 5.71), Tetanus Toxoid Vaccine (AOR = 2.43, 95% CI = 1.56, 3.77), place of delivery (AOR = 2.02, 95% CI = 1.24, 3.28), place of residence (AOR = 2.04, 95% CI = 1.33, 3.13), and household visits by health workers (AOR = 1.92, 95% CI = 1.17, 3.16), were found to be factors significantly associated with full immunization in the multivariate logistic regression analysis.

**Conclusions:**

The overall immunization coverage was found to be low. Hence, to increase the immunization coverage and reduce the incidences of missed opportunity, delivery in the health institution should be promoted, the outreach activities of the health institutions should be strengthened and greater utilization of health services by mothers should be encouraged.

## Background

Vaccination is the most effective means of combating diseases, particularly dangerous infectious diseases [1]. In 1974, the World Health Organization (WHO) launched the Expanded Program of Immunization (EPI) to make vaccines available to all children and thereby control vaccine preventable diseases worldwide [2, 3]. The vaccination of children, has led to a significant reduction in morbidity and mortality from different diseases, thereby lowering the infant mortality rate [1]. However, in 2012 the WHO revealed that around 1.5 million children worldwide died from vaccine‒preventable diseases. In the same year WHO further reported about 22.6 million children under the age of one worldwide did not receive Diphtheria-Pertussis-Tetanus Vaccine Three (DTP3) vaccine and more than 70% of these children lived in ten countries of the Democratic Republic of Congo, Ethiopia, India, Indonesia, Iraq, Nigeria, Pakistan, Philippines, Uganda and South Africa [4]. In the Sub-Saharan Africa, despite the availability of vaccines and the efforts of governments and their partners’ mortality rate of children under the age of five years remains the highest [1].

In Ethiopia, infectious and communicable diseases account for about 60‒80% of the health problems [5]. A substantial number of deaths of children under five years of age in the country is due to vaccine‒preventable diseases. The < 5 age mortality stands at 123 per 1,000 with a plan to reduce to 54 per 1,000 up to the year 2015 to meet Millennium Development Goal-4 (MDG-4) and immunization coverage will be one of the indicators to monitor the progress [5, 6]. EPI was launched in Ethiopia in1980 with the objective of achieving 100% immunization coverage of all children under two by 1990. However, in 1986, the coverage was found upon review to be 75%, and the target age group was expanded to include children under one, but the progress in increasing coverage since then has been slow [3, 7, 8]. Ethiopia strictly follows the WHO recommendations for developing countries immunization schedule for the Eight EPI vaccines for children and tetanus immunization for women of reproductive age [3]. As per the updated Ethiopian immunization policy of 2007, children under the age of one and women of 15‒49 years are the targets for the EPI vaccines. Immunization services in Ethiopia are provided free of charge in most of the health facilities as well as and in the outreach services for communities residing beyond 5 km from the health facilities [3].

Ethiopia received an award from the WHO’s Task Force on Immunization in 2004 for this best improvement in EPI coverage compared to other priority countries, and further received the Global Alliance for Vaccine and Immunization (GAVI) financial reward in 2005, 2006, and 2007 as a result of its continuous improvements in immunizing children. The improvement noted in the EPI performance in the highly populated regions has made a positive contribution to the improvement in the national DPT–Hepatitis B (HepB)–Haemophilus influenza type B three (Hib3) coverage, which met 81% of the target, as indicated in the Comprehensive Multi Year Plan (cMYP) 2006‒2010. According to the cMYP 2006‒2010 the DPT3 coverage to be attained by 2008 was 81% and the actual coverage turned to be as planned. This shows that the EPI program was implemented as per the plan and the targets set were realistic [3]. According to Ethiopia Demographic and Health Survey (EDHS), vaccination coverage increased from 14% in the 2000 to 20% in 2005. The 2011 EDHS revealed that 24% of Ethiopian children in the age group of 12–23 months received all the recommended vaccines i.e. one dose each of BCG and measles, and three doses each of DPT and polio [9]. Implementation of the Reaching Every District (RED) approach with continued GAVI support, technical and financial support from all partners in health and deployment of the health extension workers were the factors that contributed towards immunization coverage improvement [3]. However, systemic barriers related to geographical coverage still remain, requiring bridging approaches such as the enhanced outreach strategy, even as the country moves towards a more equitable geographical coverage [3].

All vaccines are provided freely and there has been improvement in the immunization coverage, however, the routine immunization coverage in Ethiopia still has not reached the target figures and realized the planned objectives. As a result, in many parts of the country, the coverage is less than the herd immunity level desired to prevent the spread of eight EPI‒targeted diseases. In particular, the Somali National Regional State (SNRS) has by far the lowest level of immunization coverage [6]. The national EPI survey conducted in 2006 revealed that the coverage for full immunization was 49.9% and that of DPT3 66.0%. These figures show significant improvement when compared to those of EDHS 2005. However, DPT3 coverage was below 80% in many of the regions and the lowest (23.3%) in the SNRS [10]. The Ethiopian annual performance report of the Health Sector Development Program III (HSDP-III) revealed that, DPT3 and measles, and full immunization coverage of SNRS were 14.6%, 18.5%, and 9.5%, respectively figures much lower than the national standard for immunization of 73%, 65%, and 53% [6].

In Ethiopia, uneven distribution, poor skill mix and high attrition of trained health professionals remain the major concern, impeding transfers of competency [5]. Similarly, immunization coverage varies significantly from region to region, ranging from only 9% of children fully vaccinated in Affar region to 79% in Addis Ababa. Over 90% of age one children in Southern Nations Nationalities Peoples’ Region (SNNPR) are immunized with DPT3 while the figure dramatically drops to less than 50% in Somali, Afar, and Gambella regions [3, 9]. The pastoralist communities in Somali, Afar, Gambella and other areas have very low routine immunization coverage and the traditional static and outreach strategies are not working well there. Thus, there is a need to design special immunization service delivering strategies for these regions [3]. Enhanced routine immunization (ERI) was conducted in some districts of SNRS in 2007 with good results. In Jigjiga zone, two districts conducted ERI and the coverage of Penta 3 increased from an average of 24% to 74% after two rounds of ERI [9]. As per the 2008 coverage report, OPV3 coverage reached 44% in Gambella, 36% in Benishangul/Gumuz and 29% in the SNRS [3]. According to the annual performance report of HSDP-III (2009) SNRS had by far the lowest level of immunization in Ethiopia compared to the other administrative regions [6]. So assessing the factors that contribute to low coverage is important in order to devise evidence-based strategies/polices that would raise the overall immunization coverage which in turn reduce the infant and child mortality.

## Methods

### Study design

A community-based cross-sectional study was conducted to assess the factors associated with the immunization coverage of children aged between 12-23 months in Jigjiga district from April 10 to May 5, 2011.

### Study area

The study was conducted in Jigjiga district, which is one of the six districts of the Jigjiga Administrative Zone and the capital city of SNRS. It is located 632 km east of Addis Ababa, the capital of Ethiopia. The district has a population of 277,560 of which 151,684 is rural and the rest urban [11] and about 15.3% are children under five of age. There is one hospital, six health centers and 28 health posts which routinely provide immunization to children under five in the district.

### Source population and study population

The source population comprised all the households in Jigjiga district having children aged between 12‒23 months, whereas the study populations comprised all mothers/caretakers with one or more child aged 12‒23 months old, that were selected by simple random sampling method from the source population in urban and rural wards in the district.

### Sample size determination

The sample size was calculated using two population proportion formulas on the basis of the study of full immunization coverage conducted in northern Ethiopia district. The immunization coverage was found to be 80% in the rural areas and 67.5% in urban areas [12].

N = (p1q1 + p2q2)F(α, β)/(*p*1 - *p*2)^2^ = 275.3, then adding 10% non respondent, the final sample size was 303 households for each urban and rural wards. α = the level of significance was 5%, and β = the power of the study 90%.

### Sampling procedure

The whole Jigjiga district was first stratified into 10 urban and 32 rural wards. Out of the two strata, two urban and four rural wards were randomly selected by lottery. A census was carried out in selected urban and rural wards to identify the households with children aged between 12 and 23 months. A sampling frame was prepared after the identification of households with children eligible for the study. The numbers were allocated proportionally to the selected wards on the basis of the population size of the children, followed by simple random sampling to select the individual child. For those households having more than one child, one child per household was randomly selected by lottery.

### Data collection procedure and quality management

The questionnaires were prepared in English then translated into Somali (native language) and back into English to ensure consistency. The questionnaire was pre-tested using 5% of the sample size, and some modifications were made on the basis of pre-test. The data were collected through face-to-face interview with the mothers/caregivers based on the structured questionnaire and through a review of the vaccination cards. Vaccination cards were reviewed and the mothers/caregivers were inquired for tracing the childrens’ immunization history. Ten trained diploma nursing students from the Jigjiga Health Sciences College participated in the data collection. Completeness and consistency of the collected data were checked each day by the principal investigator.

### Data processing and analysis

The collected data were cleaned, entered and analyzed using Statistical Package of Social Sciences (SPSS) version 16. The dependent variable was dichotomized into fully vaccinated and not fully vaccinated (unvaccinated and partially vaccinated). The full immunization status of the children assessed from the vaccination cards as well as the mother’s/caregiver’s responses were used in the analysis of both bivariate and multiple logistic regression. To identify the factors associated with the immunization status of children, bivariate and multiple logistic regression analyses were worked out and *p*-value < 0.05 was considered to indicate statistical significance. In this study, the Hoshmer and Lemeshow’s goodness-of-fit was used to assess whether a multiple logistic regression model was fit. A model with *p*-value > 0.05 of Hoshmer and Lemeshow’s test was considered as fit for multiple logistic regressions.

### Operational definitions of the terms used in the study

The following operational definitions were used:

#### Fully immunized

A 12‒23 months old child who received one dose of BCG and measles, and three doses of pent/OPV before his/her first birthday.

#### Partially immunized

A 12‒23 months old child who received at least one vaccine, but not all the EPI vaccines.

#### Unimmunized

A 12‒23 months old child who did not receive any of the EPI vaccines.

#### Not fully immunized

A combination of partially vaccinated and unvaccinated children.

#### Immunization coverage by card

The vaccination coverage calculated with numerator based only on card documentation, excluding from the numerator those vaccinated by history.

#### Immunization coverage by history

The vaccination coverage calculated with numerator based only on mother’s/caregiver’s report.

### Ethical clearance

Ethical clearance was obtained from the Institutional Review Board of the Institute of Public Health, University of Gondar. Collaboration letter was obtained from SNRS Health Bureau, Jigjiga District Health Office and wards administration. Study participants were briefly informed about the objective and the significance of the study and finally their consent was obtained. The respondents were told that they could withdraw from the study at any time they wanted. Confidentiality of the data was maintained throughout the study period and the names of the study participants were omitted from the questionnaires.

## Results

### Characteristics of study participants

A total of 582 mothers/caregivers who had 12‒23 months old children participated in the study. The majority (92.4%) of mothers/caregivers were married and 87% were illiterate. Table 1 shows the socio-demographic characteristics of the study participants. About 52% of the children were male and the rest were female. Seventy five percent of children were in the age group of 15 to 23 months while the remaining 25% were in the age group of 12-14 months. Table 2 shows the selected characteristics of the children. Nearly an equal number of urban (49.8%) and rural (50.2%) mothers/caregivers were participated in the study. The majority (75.6%) of mothers/caregivers reported that health workers did not come to their homes for child vaccination. Table 3 presents the selected characteristics of the study participants.

**Table 1:** **Socio-demographic characteristics of mothers/caregivers in Jigjiga District**

Variable	Number (n = 582)	Percent (%)
Age (in years)		
15-19	92	15.8
20-24	190	32.6
25-29	173	29.7
30 ≥	127	21.8
Marital status		
Married	538	92.4
Divorced	36	6.2
Widowed	8	1.4
Educational status		
Illiterate	510	87.6
Literate	72	12.4
Ethnicity		
Somali	536	92.1
Non-Somali	46	7.9
Occupation		
Government employee	3	0.5
Private company/organization	3	0.5
Trader	8	1.4
House wife	512	88.0
Student	11	1.9
Others**	45	7.7
History of Antenatal care attendance		
Yes	253	43.5
No	329	56.5
Tetanus toxoid vaccine		
Yes	242	41.6
No	340	58.4

Key: **: milk seller, fire wood seller and daily laborer.

**Table 2:** **Selected characteristics of 12-23 months children by residence in Jigjiga District**

Variable	Urban (n = 290)	Rural (n = 292)	Total
	n (%)	n (%)	n (%)
Sex of the child			
Male	149 (48.7)	157 (51.3)	306 (52.6)
Female	141 (51.1)	135 (48.9)	276 (47.4)
Child’s birth order			
First	52 (41.3)	74 (58.7)	126 (21.6)
Second	66 (50.0)	66 (50)	132 (22.7)
Third	50 (45.9)	59 (54.1)	109 (18.7)
Fourth or more	122 (56.8)	92 (43.2)	205 (36.9)
Child’s birth place			
Health institution	77 (49)	80 (51)	157 (27)
Home	213 (50.1)	212 (49.9)	425 (73)

**Table 3:** **Selected characteristics of study participants by residence in Jigjiga District (n = 290 Urban and n = 292 Rural)**

Variable	Urban n (%)	Rural n (%)	Total n (%)
Household income (in ETB)			
<300	33 (30.8)	74 (69.2)	107 (18.4)
301-600	94 (45.9)	111 (54.1)	205 (35.2)
601-900	79 (60.8)	51 (39.2)	130 (22.3)
>900	84 (60)	56 (40)	140 (24.1)
Family size			
Three	52 (42.6)	70 (57.4)	122 (21)
Four	43 (44.3)	54 (55.7)	97 (16.7)
Five	71 (52.6)	64 (47.4)	135 (23.2)
Six or more	124 (54.4)	104 (45.6)	228 (39.2)
Travelling time to a health facility			
<30 minutes	74 (76.3)	23 (23.7)	97 (16.7)
30-60 minutes	87 (55.1)	71 (44.9)	158 (27.1)
>60 minutes	129 (39.4)	198 (60.6)	327 (56.2)
Waiting time at health facility			
1-10 minutes	38 (73.1)	14 (26.9)	52 (8.9)
11-20 minutes	57 (51.8)	53 (48.2)	110 (18.9)
21-30 minutes	51 (45.9)	60 (54.1)	111 (19.1)
>30 minutes	144 (46.6)	165 (53.4)	309 (53.1)
Taking the child to the health facility while sick for medical reason			
Yes	176 (53)	156 (47)	332 (57)
No	114 (45.6)	136 (54.4)	250 (43)
Provision of health education at health facility			
Yes	50 (57.5)	37 (42.5)	87 (14.9)
No	128 (51.8)	119 (48.2)	247 (42.4)
Home visits by health worker			
Yes	82 (57.1)	60 (42.9)	142 (24.4)
No	208 (47.3)	232 (52.7)	440 (75.6)

### Status of immunization

**Figure 1:**
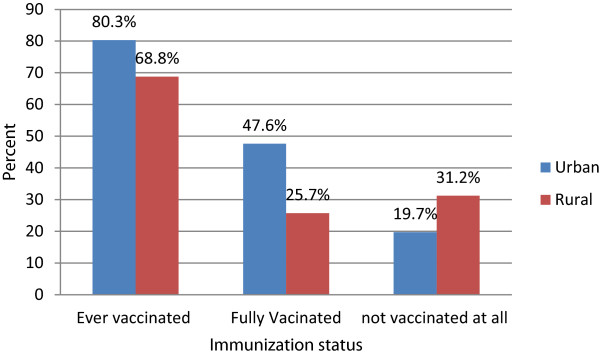
**Immunization status of 12-23 months children by residence in Jijiga District.**

A review of the vaccination cards and mothers’/caregivers verbal responses revealed that out of the 582 children, 74.6% were ever vaccinated, 36.6% fully vaccinated and 25.4% not vaccinated at all. Around 80.3% of urban and 68.8% of rural children were ever vaccinated. About 47% urban and 25.7% rural children were fully vaccinated, and 19.7% of urban and 31.1% of rural children were not vaccinated at all. Figure 1 shows the immunization status (vaccination card + mothers’/caregivers’ verbal responses) of the children by residence.

According to the review of the vaccination cards, the highest percentage of vaccination was 41.8% for BCG, while the lowest percentage was 24.9% for measles. From Table 4, we can observe that the percentage of children receiving DPT and polio vaccines shows a decrease from early to late vaccines. The coverage of DPT1 is higher than DPT 2 and the coverage of DPT 2 is higher than DPT 3. The percentage of children receiving each polio vaccine shows a reduction i.e. 41%, 33.8% and 27.4% of polio 1, polio 2, and polio 3, respectively. The main reasons given by mothers/caregivers why their children were not fully vaccinated were lack of information, lack of motivation, and obstacles to immunization (Table 5).

**Table 4:** **Vaccination coverage of 12-23 months children by residence based on the vaccination card and report from the mother/caregiver in Jigjiga District (n = 290 Urban and n = 292 Rural)**

Vaccines	Card			Mothers/caregivers report		
	Urban n (%)	Rural n (%)	Total n (%)	Urban n (%)	Rural n (%)	Total n (%)
BCG	128 (44.1)	115(39.4)	243(41.8)	105 (36.2)	86 (29.45)	191 (32.8)
OPV0	26 (8.9)	35 (11.9)	61 (10.4)	10 (3.45)	14 (4.79)	24 (4.1)
OPV1	126 (43.4)	113 (38.7)	239 (41.1)	100 (34.48)	86 (29.45)	186 (31.9)
OPV2	109 (37.65)	88 (30.1)	197 (33.9)	76 (26.2)	60 (20.55)	136 (23.3)
OPV3	97 (33.4)	63 (21.6)	160 (27.5)	60 (20.68)	37 (12.67)	97 (16.6)
Pentavalent1	126 (43.4)	113 (38.7)	239 (41.1)	100 (34.48)	86 (29.45)	186 (31.9)
Pentavalent2	109 (37.65)	88 (30.1)	197 (33.9)	76 (26.2)	60 (20.55)	136 (23.3)
Pentavalent3	97 (33.4)	63 (21.6)	160 (27.5)	60 (20.68)	37 (12.67)	97 (16.6)
Measles	91 (31.3)	54 (18.55)	145 (24.9)	47 (16.2)	21 (7.2)	68 (11.68)
Fully vaccinated	91 (31.3)	54 (18.55)	145 (24.9)	47 (16.2)	21 (7.2)	68 (11.68)

**Table 5:** **Reasons of mothers/caregivers for not fully vaccinating children in the Jigjiga District**

Reasons	Number (%)
**Lack of information**	
Unaware the need of immunization	3 (0.5)
Unaware the need to return for 2nd and 3rd dose	47 (8.1)
Unaware time of measles vaccine	3 (0.5)
Fear of side reactions	2 (0.3)
**Lack of motivation**	
Don't believe in vaccination	12 (2.1)
Busyness due to work load	137 (23.5)
Thinking that child will vaccinate in the other time	8 (1.4)
Long queue in the vaccination center	3 (0.5)
Forgetting vaccination date	46 (7.9)
**Obstacles**	
Absence of vaccinator	10 (1.7)
Vaccine centre was too far	38 (6.5)
There was an abscess at the place of vaccine	9 (1.5)
The child faced difficulty after receiving vaccine	11 (1.9)
The child was sick	16 (2.7)
Mother was sick	12 (2.1)
Mothers/gargivers thought the vaccinator would cometo home	11 (1.9)

### Factors predicting child's immunization status

In the multiple logistic regression analysis, maternal age and literacy, place of residence, TT vaccine status, place of delivery and household visit by health workers were found to be the most important predictors of completing immunization (according to the vaccination cards plus mothers/care givers response) (*p* <0.05) (Table 6).

**Table 6:** **Multiple logistic regression analysis of factors predicting fully vaccinated status (vaccination cards plus mothers recall) of 12-23 months children in the Jigjiga District**

Variables	Immunization status			
	Fully vaccinated	Not vaccinated	95% CI COR	95% CI AOR
	n (%)	n (%)		
Age of the mother				
15-19	14 (15.2)	78 (84.8)	1.00	1.00
20-24	61 (32.1)	129 (67.9)	2.63 (1.44,3.63)	2.19 (1.26,3.83)*
25-29	72 (41.6)	101 (58.4)	3.97 (0.96,2.41)	1.31 (0.76,2.26)
≥ 30	66 (52)	61 (48)	6.03 (3.09,11.74)	3.79 (1.76,8.16)*
Mother’s education				
Literate	46 (63.9)	26 (36.1)	3.63 (2.17,6.08)	3.06 (1.64,5.71)*
Illiterate	167 (32.7)	343 (67.3)	1.00	1.00
Place of residence				
Urban	138 (47.6)	152 (52.4)	2.63(1,85,3.73)	2.04 (1.33,3.13)*
Rural	75 (25.7)	217 (74.3)	1.00	
TT vaccine				
Yes	139 (57.4)	103 (42.6)	4.85 (3.38,6.97)	2.43 (1.56,3.77)*
No	74 (21.8)	266 (78.2)	1.00	1.00
Place of delivery				
Health institution	88 (56.1)	69 (43.9)	3.06 (2.10,4.47)	2.02 (1.24,3.28)*
Home	125 (29.4)	300 (70.6)	1.00	1.00
Household visit by health workers				
Yes	73 (52.1)	67 (47.9)	2.35 (1.60,3.46)	1.92 (1.17,3.16)*
No	140 (31.7)	302 (68.3)	1.00	1.00

Key: CI: confidence interval, *: statistically significant at p < 0.05, 1.00: reference group, COR: Crude Odd Ratio, AOR: Adjusted Odd Ratio.

## Discussion

Overall, the immunization coverage was low, as by convention the immunization coverage of DPT3 or in our case Pentavalent 3 (as per the vaccination cards) was 27.5% (Table 4) which is higher than the percentage of coverage (10%) reported from the pastoral community of Amibara district, ANRS [13] and much lower than those from the northern rural district of Ethiopia (92.7%) [12]; Ambo Woreda, in Central Ethiopia (35.6%) [14]; Kenya (88%) [15] and rural Nigeria (80.8%) [16]. This might be due to low access to immunization services, inadequate knowledge of mothers/caregivers and high dropout rates in the study area. Another reason could be the high percentage of missed opportunity (74%), which is higher than those in Wango district of Ethiopia (46.3%) [17] and Mozambique (25.7%) [18]. Proper screening of immunization status might not have been done by the healthcare personnel when mothers/caretakers came to health facilities with their children for preventive and curative services.

The study reveals that out of the total number of children studied, fully vaccinated ones (as per the card and mothers/caregivers response) constituted only 36.6%, and were more numerous in urban (47.50%) than in rural (25.7%) settings. The percentage of fully vaccinated children was also found to be higher than that among pastoral community of Amibara district, ANRS (8.3%) [13] and almost close to that reported from Ambo Woreda of Central Ethiopia (36%) [14]. But it was lower than that reported from the northern rural district of Ethiopia [75.5% (80% urban and 67.5% rural)] [12]. The reasons for such differences in immunization coverage within the same country might be better access to immunization services in the northern rural district of Ethiopia as evinced by the 97.3% coverage of OPV1/DPT1 there [12]. The percentage of fully immunized children was also lower than that in Istanbul (84.5%) [19] and Mali (59.9%) [20], this might be due to low educational status of mothers/caretakers, high defaulters, inadequate knowledge and low health service utilization in the study area. About 25.4% of children were without immunization of any type, a very high figure when compared to only 3.2% in Istanbul [19]. The reason could be that the mothers/caretakers in the study area were not of aware the importance of immunization, and the place and/or time of immunization, feared side effects of vaccines or had wrong ideas about contraindications. For instance, the mothers/caregivers of unimmunized children cited reasons such as unawareness of the need for immunization, lack of confirmed information and lack of time to get their children vaccinated reasons that are same as those cited by mothers/caregivers in Mali [20]. The additional reasons as reported by mothers/caregivers in Mozambique were that the waiting time was long, there were no healthcare personnel at health facilities, they were unaware of or forgot the day of vaccination, and no vaccines could be given due to child sickness [18].

Thirty eight percent of the children in this study were partially immunized. This could be due to the fact that many children not subjected to follow‒up vaccination in later months, which may be due to low access to immunization services, as evidenced by low Pentavalent1 coverage and high missed opportunity. Reasons mentioned by the mothers/caregivers of such partially immunized children who defaulted on vaccination were lack of information, motivation and obstacles. Similarly, obstacles (67%), lack of information (19%) and lack of motivation (14%) were the commonest reasons cited for partial vaccination of children in Amibara district [13].

Upon the review of vaccination cards, a higher immunization coverage was found for pentavalent1 (41.1%) and a lower one for pentavalence3 (27.5%). This difference could be due to the absence of vaccinators at health facilities, low utilization of maternal and child health care services. The dropout rate of pentavalent1 to pentavalent3 was 33.1% which is less than 43% as reported in the EDHS 2011 [21], but higher than 22.6% reported from Kenya [15]. The dropout rate from BCG to measles in the present study was 40.3% which is comparatively higher than that in other districts of Ethiopia [12, 17]. The difference might be due to high percentage of defaulters, lack of local motivators for reminding/tracking and absence of health workers at health facilities.

Maternal education positively influenced the completion of child immunization. This can be seen in the difference between the percentage of literate (63.9%) and illiterate (32.7%) mothers fully vaccinating their children. Literate mothers were 3.06 times more likely to fully vaccinate their children than illiterate ones (AOR = 3.06, 95% CI = 1.64, 5.71). In other similar studies, maternal education has been reported as a predictor of child immunization completion [12, 13, 15, 16, 22]. This may be due to the literate mothers having better knowledge of vaccine-preventable diseases and recognizing the importance of vaccination.

Full immunization was found to have a significant association with the outreach activities of the health institutions. The probability of a child to be fully vaccinated was higher when the health workers visited homes for vaccination (AOR = 1.92, 95% CI = 1.17, 3.16). This is in line with the study done in Murshidabad district of West Bengal, India [22]. Household visits contributed to higher rates of immunization, especially in case of illiterate mothers, mothers who had delivered outside health facilities and mothers who had no health education on immunization [23, 24].

The rate of complete vaccination of children increases with the age of their mothers, as 15.2% of mothers between 15 and 19 years of age, 32.1% between 20 and 24 years, 41.6% between 25 and 29 years and 52% of 30 years or more were found to have vaccinated their children fully. Mothers/caregivers of 30 years or more were 3.79 times more likely to fully vaccinate their children than mothers/caregivers aged between 15 and 19 years (AOR = 3. 79, 95% CI = 1. 76, 8.16). Old-aged mothers/caregivers were more likely to have their children fully immunized than teenage mothers/caregivers [25]. In Bangladesh, the mother’s/caregivers’ age was found to be the most important predictor for full immunization of children [26].

In agreement with the studies carried out in Amibara district of Ethiopia [14] and Bangladesh [26], the present study also revealed that mother’s/caregivers’ TT immunization acceptance was found to be the most important predictor for full immunization of children. Mothers/caregivers who took any TT vaccine during pregnancy were 2.43 times more likely to fully vaccinate their children than mothers/caregivers who took none (AOR = 2.43, 95% CI = 1.56, 3.77). In another study in Bangladesh, mothers/caregivers who did not receive TT vaccination during pregnancy were 70% less likely to have their children fully immunized than those who received it [25].

Regarding the place of delivery, the study showed that 56.1% of children born in health care facilities were fully immunized as against 29.4% of children born at homes. Further, children born in health institutions were 2.02 times more likely to be vaccinated completely than those born at home (AOR = 2.02, 95% CI = 1.24, 3.28). Similarly, the studies conducted in Amibara district and Ambo district within the country, and in Nigeria showed that children born in health facilities were more likely to complete vaccination than those born at homes [13, 14, 27]. The reason might be that government hospitals and healthcare centers, as matter of policy provide BCG and OPVO vaccination, as well as health education about benefits of vaccination to the mothers/caregivers after delivery [27].

## Conclusions

Our study, upon investigation of low immunization coverage and high dropout rate found that the area of residence; the mother’s/caregivers’ age at the time of delivery, her educational status, and the status of her TT vaccination; the place of delivery; household visits by health workers all were important predictors for complete immunization.

Further, our study makes several crucial recommendations like to prevent mothers/caregivers from defaulting on immunization of their children, health workers must regularly visit the households with children aged between 12 and 23 months, in order to provide health education and vaccines as also track defaulters. Such children when taken by mothers/caretakers to health facilities for any purpose should be screened to check their immunization status in order to prevent missed opportunities. Outreach sites should be established to encourage urban health extension workers to reach out to mothers/caregivers who are busy with housework and hence ignore or default on immunization. Training in immunization and communication skills should be provided to health workers so that they can convince mothers/caregivers of the benefits of vaccination and encourage them to visit health facilities.

### Limitation

In Ethiopia, majority of the population lives in rural areas. However, due to financial constraints we failed to take many study participants from rural areas and hence simply took an equal number of study participants from both urban and rural areas.
